# Binding of [^18^F]AV1451 in post mortem brain slices of semantic variant primary progressive aphasia patients

**DOI:** 10.1007/s00259-019-04631-x

**Published:** 2019-12-18

**Authors:** Jolien Schaeverbeke, Sofie Celen, Julie Cornelis, Alicja Ronisz, Kim Serdons, Koen Van Laere, Dietmar Rudolf Thal, Thomas Tousseyn, Guy Bormans, Rik Vandenberghe

**Affiliations:** 1 Laboratory for Cognitive Neurology, Department of Neurosciences, KU Leuven, Herestraat 49, 3000 Leuven, Belgium; 2 Laboratory of Radiopharmaceutical Research, KU Leuven, Herestraat 49, 3000 Leuven, Belgium; 3 Laboratory for Pathology, Department of Imaging and Pathology, KU Leuven, Herestraat 49, 3000 Leuven, Belgium; 4 Leuven Brain Institute, Herestraat 49, 3000 Leuven, Belgium; 5 Nuclear Medicine and Molecular Imaging, University HospitalsLeuven, Herestraat 49, 3000 Leuven, Belgium; 6 Pathology division, Department of Pathology, University Hospitals Leuven, Herestraat 49, 3000 Leuven, Belgium; 7 Neurology division, Department of Neurology, University Hospitals Leuven, Herestraat 49 box 7003, 3000 Leuven, Belgium

**Keywords:** Neurodegeneration, Tauopathy, Frontotemporal, Aphasia, TDP-43, AV1451, THK5351

## Abstract

**Purpose:**

In vivo tau-PET tracer retention in the anterior temporal lobe of patients with semantic variant primary progressive aphasia (SV PPA) has consistently been reported. This is unexpected as the majority of these patients have frontotemporal lobar degeneration TDP (FTLD-TDP).

**Methods:**

We conducted an in vitro [^18^F]AV1451 autoradiography binding study in five cases with a clinical diagnosis of SV PPA constituting the range of pathologies (i.e., three FTLD-TDP, one Alzheimer’s disease (AD), and one Pick’s disease (PiD)). Binding was compared with two controls without neurodegeneration, two typical AD, one corticobasal syndrome with underlying AD, and one frontotemporal dementia behavioral variant with FTLD-TDP. The effect of blocking with the authentic reference material and with the MAO-B inhibitor deprenyl was assessed. Immunohistochemistry was performed on adjacent cryosections.

**Results:**

Absence of specific [^18^F]AV1451 binding was observed for all three SV PPA FTLD-TDP cases. The absence of binding in controls as well as the successful blocking with authentic AV1451 in cases with tauopathy demonstrated specificity of the [^18^F]AV1451 signal for tau. The specific [^18^F]AV1451 binding was highest in AD, followed by PiD. This binding colocalized with the respective tau lesions and could not be blocked by deprenyl. Similar pilot findings were obtained with [^18^F]THK5351.

**Conclusion:**

In vitro autoradiography showed no [^18^F]AV1451 binding in SV PPA due to FTLD-TDP, while specific binding was present in SV PPA due to AD and PiD. The discrepancy between in vitro and in vivo findings remains to be explained. The discordance is not related to [^18^F]AV1451 idiosyncrasies as [^18^F]THK5351 findings were similar.

**Electronic supplementary material:**

The online version of this article (10.1007/s00259-019-04631-x) contains supplementary material, which is available to authorized users.

## Introduction

The semantic variant of primary progressive aphasia (SV PPA), also known as semantic dementia [1], is a language-based neurodegenerative disorder which presents as a fluent, anomic aphasia with single-word comprehension problems and generally progressive loss of vocabulary and non-verbal knowledge [1, 2]. SV PPA has been associated with bilateral atrophy of the ventrolateral anterior temporal lobe, which is usually more prominent in the dominant language hemisphere [3]. In 69–83% of cases, the underlying pathology is frontotemporal lobar degeneration (FTLD) transactive response DNA-binding protein TAR DNA-binding protein-43 (TDP-43) type C pathology [4, 5]. Alzheimer’s disease (AD) pathology occurs in up to 20% of clinical cases and more rarely Pick’s disease (PiD) is seen as underlying pathology [6]. Currently, a positron emission tomography (PET) tracer to visualize TDP-43 in vivo is not available yet. However, first-generation tau-PET tracers such as [^18^F]AV1451 [7] and [^18^F]THK5351 [8] have consistently shown strong in vivo tracer retention in the anterior temporal lobe of SV PPA patients [9–16]. At the individual patient level, evidence for consistently elevated [^18^F]AV1451 binding has been found in the anterior temporal lobe of all seven SV PPA cases and all seven “right” semantic dementia cases [10, 11]. In a separate study, this pattern was also observed in all 13 SV PPA cases who were amyloid-negative [14]. Elevated binding in this peak region has also been consistently observed with [^18^F]THK5351 across SV patients [13, 15, 16]. These findings are surprising since the vast majority of SV PPA patients have underlying TDP-43 proteinopathy [5] and are not expected to show [^18^F]AV1451 binding in the anterior temporal lobe as the ligand has selectivity for tau over amyloid (29 fold) [7].

In vitro binding studies with fluorine-18 labeled and tritiated AV1451 on sections with FTLD pathology have shown heterogeneous results [12, 17–21]. [^3^H]AV1451 autoradiography indicated no binding to TDP-43 type C in SV PPA nor to C9orf72 gene mutation carriers with underlying TDP-43 type B pathology [12]. Similar absence of binding was observed with [^18^F]AV1451 phosphor screen autoradiography in TDP-43 type A [22]. Absence of in vitro binding in cases with underlying TDP-43 has also been reported by an independent study [21]. On the other hand, Sanders et al. and Lowe et al. suggested minimal binding to TDP-43 and strong binding to tau, in particular to AD tau [20, 23].

We hypothesize that the elevated in vivo [^18^F]AV1451 PET signal consistently observed in the anterior part of the inferior temporal/occipitotemporal gyrus in SV PPA is possibly related to TDP-43 binding or to MAO-B affinity. As indicated by a recent [^3^H]AV1451 autoradiography study on brain homogenates devoid of tau pathology, the high in vivo [^18^F]AV1451 retention in SV PPA might be related to binding to monoamine oxidase (MAO) A and B enzymes [24]. [^18^F]THK5351 competition autoradiography also showed affinity for MAO-B on AD sections [25]. MAO-B is abundantly expressed on reactive astrocytes which accompany neurodegeneration [26]. Expression of MAO-B by reactive astrocytes can be visualized by specific MAO-B PET tracers including [^11^C]deuterium-L-deprenyl [27].

Based on these findings, we pursued the hypothesis that [^18^F]AV1451 and [^18^F]THK5351 binding in SV PPA is related to affinity for MAO-B enzymes. We conducted an in vitro autoradiography binding study with [^18^F]AV1451 in cases with a clinical diagnosis of SV PPA, constituting the range of underlying pathologies (i.e., TDP-43 type C, AD, and PiD) and compared binding to controls and cases with typical AD, corticobasal syndrome (CBS) due to neuropathological AD, and FTD behavioral variant (FTD-bv) due to TDP-43 type C. Coincubation with the MAO-B inhibitor deprenyl (selegiline) was performed in all cases to study MAO-B binding. A subset of cases was assessed with [^18^F]THK5351 to assess translatability of findings to another first-generation tau-PET tracer.

## Materials and methods

### Study subjects

Human brain tissue was obtained from the University Hospitals Leuven (UZ)/KU Leuven Brain BioBank, hosted at UZ Leuven (Leuven, Belgium). Autopsies were performed in accordance with Belgian law and this study has been approved by the Biobank Board KU/UZ Leuven and by the Ethics Committee KU Leuven from the local ethical committee in Leuven/Belgium (No. s52791, s59292, s59295). The study protocol was in accordance with the ethical standards from the Declaration of Helsinki. All patients have been repeatedly examined during life by an experienced neurologist (RV) at UZ Leuven (Neurology Department, Leuven, Belgium). Cases have been selected based on both the clinical syndrome and the neuropathological diagnosis: cases with a clinical diagnosis of SV PPA, constituting the range of underlying pathologies (i.e., TDP-43 type C, AD, and PiD) were selected and compared with controls and cases with typical AD, corticobasal syndrome (CBS) due to neuropathological AD, and FTD-bv due to TDP-43 type C. For patient selection, cases of familial AD, Down syndrome, Creutzfeldt–Jakob disease, familial cerebral amyloid angiopathy (CAA), inflammatory diseases of the brain or the vessels, large brain tumors, vascular malformations, and severe head traumata were excluded, to ensure that these factors did not interfere with the assessment of vascular dementia or AD as the causes of neurological symptoms. Five cases had a clinical diagnosis of SV PPA according to published clinical criteria at the time of diagnosis [2], two had a clinical diagnosis of AD [28], one case was diagnosed as CBS [29], and one as FTD-bv [30] (Table 1). Two cases were cognitively intact older non-AD/non-FTLD controls of which one had Guillain–Barre syndrome and the other died from glioblastoma multiforme WHO grade IV in the left temporo-occipital region (size: 4.5 × 4 × 3.5 cm). For the current experiment, brain tissue was taken from the right, fresh frozen, unfixed hemisphere, which was free of tumor tissue. All cases have been retrospectively assigned a clinical dementia rating score (CDR) (Table 1).

**Table 1:** Subject characteristics

Subject	Clinical diagnosis	Pathological diagnosis	CDR score	Age at death (yrs)	Sex	Braak NFT stage	Amyloid phase	Inferior temporal gyrus/occipitotemporal gyrus					
								AD p-tau	FTLD p-tau	FTLD pTDP-43	Astro-gliosis	Micro- gliosis	Iron
1	CTRL	GBS, PART	0	54	M	I	0	1	0	0	1	1	No siderophages
2	CTRL	Glioblastoma, PART	0	68	M	I	0	1	0	0	1	1	No siderophages
3	AD	AD	2	72	M	VI	5	4	0	0	2	2	No siderophages
4	AD	AD	2	87	M	VI	5	4	0	0	2	2	No siderophages
5	CBS	AD	3	54	F	VI	5	4	0	0	2	2	No siderophages
6	SV PPA	AD	1	69	M	VI	5	4	0	0	2	2	No siderophages
7	SV PPA	PiD	3	66	M	0	0	0	3	0	2	2	No siderophages
8	SV PPA	FTLD-TDP: type C AGD	2	81	F	II	2	0	3	3	3	2	No siderophages
9	SV PPA	FTLD-TDP: type C	2	69	F	I	0	1	0	2	2	2	No siderophages
10	SV PPA	FTLD-TDP: type C	3	79	M	I	0	1	0	1	2	2	No siderophages
11	FTD-bv	FTLD-TDP: type C AD, mild LBD	3	72	F	III	4	3	0	2	2	2	No siderophages

Clinical, demographic, and pathological data. Pathology: Braak NFT stage (0–VI) [31] and amyloid phase (0–5) [33]. Range of other pathologies is expressed as a semi-quantitative score: 0, no pathology; 1, mild (up to 3 lesions/HPF); 2, moderate (4–20 lesions/HPF); 3, severe (more than 20 lesions/HPF); 4, lesions clearly visual by the naked eye; For astrogliosis (GFAP) and microgliosis (CD68), the semi-quantitative score ranges between 0 and 3. Iron content is visualized by Perl’s staining. *AD*, Alzheimer’s disease; *AGD*, argyrophilic grain disease; *CBS*, corticobasal syndrome; *CDR*, clinical dementia rating scale; *CTRL*, control; *F*, female; *FTD-bv*, frontotemporal dementia behavioral variant; *FTLD-TDP*, frontotemporal lobar degeneration transactive response DNA-binding protein pTDP-43; *GBS*, Guillain-Barre syndrome; *LBD*, Lewy Body disease; *M*, male; *NFT*, neurofibrillary tangle; *PART*, primary age-related tauopathy; *PiD*, Pick’s disease; *SV PPA*, semantic variant primary progressive aphasia

### Neuropathology

For neuropathological diagnosis, including the detection of large and lacunar infarcts, microinfarcts, hemorrhages, and microbleeds, paraffin-embedded sections were stained with hematoxylin and eosin (H&E). Neurofibrillary tangles (NFTs), neuropil threads, and neuritic plaques were assessed using the Gallyas silver method [31] as well as using an antibody directed against abnormal phosphorylated τ-protein (AT8, 1:1000, Ser202/Thr205, PierceThermoScientific, Waltham, MA, USA) [32]. AT8 staining was also used to assess binding to FTLD-tau (corticobasal degeneration (CBD), progressive supranuclear palsy (PSP), argyrophilic grain disease (AGD), NFT-predominant dementia, and PiD). Aβ deposits were detected with an antibody raised against Aβ_17–24_ (4G8, 1:5000, formic acid pretreatment, Covance, Dedham, USA). An antibody raised against the phosphorylated TDP-43 (polyclonal rabbit-pS409/410–2, 1:5000, low pH citrate pretreatment, Cosmo Bio Co., Ltd., Tokyo, Japan) was used to detect pTDP-43 aggregates and to differentiate microinfarcts in the hippocampus from TDP-43-related hippocampal sclerosis. These primary antibodies were marked with a biotinylated secondary antibody. The secondary antibodies were visualized with the EnVision Flex HRP (Dako, Agilent) and 3,3-diaminobenzidine (DAB). Immuno-labeled paraffin sections were counterstained with hematoxylin. All tissue sections were viewed with an Olympus BX 51 or a Leica DMLB 2 light microscope by a neuropathologist (DRT). Digital photographs were taken with a Leica DC 500 or a Leica DCC 290 camera.

For each case, a Braak NFT stage [31], amyloid phase [33], Consortium to Establish a Registry for Alzheimer’s disease (CERAD) neuritic plaque score [34, 35], and FTLD-TDP subtype [36, 37] were assigned in accordance with published guidelines. Neuropathologically, of the SV PPA cases, three had FTLD-TDP type C, one had neuropathological AD, and one had underlying PiD. The two typical AD as well as the CBS case had underlying AD pathology (Table 1). The FTD-bv case was diagnosed with TDP-43 type C pathology. The two negative controls had primary age-related tau pathology (PART) in the medial temporal lobe [38], but no other neurodegenerative or cerebrovascular pathology (Table 1). All cases were included in the [^18^F]AV1451 autoradiography binding study. A subset of these cases (case 4, 6, 8, and 11) (Table 1) was included in the [^18^F]THK5351 autoradiography binding study to assess translatability of findings to another first-generation tau-PET tracer.

### Radiosynthesis of [^18^F]AV1451 and [^18^F]THK5351

GMP-compliant automated production of [^18^F]AV1451 was performed in a Trasis All-In-One® radiosynthesizer (Trasis, Ans, Belgium) by a nucleophilic fluorination of the AV1622 precursor (5-(5-(tert-butoxycarbonyl)-5H-pyrido[4,3-b]indol-7-yl)-N,N,N-trimethylpyridin-2-aminium 4-methylbenzenesulfonate, AVID Radiopharmaceuticals, Philadelphia, PA, USA). [^18^F]fluoride was produced, separated from the ^18^O-water, eluted from the anion exchange cartridge and azeotropically dried as described by Declercq et al. [39] with some minor modifications: synthesis was performed fully automated using a disposable cassette as compared with the previous report where radiosynthesis was done in remote controlled home-made modules. A volume of 0.85 mL (instead of 0.75 mL) Kryptofix/K_2_CO_3_ solution and 0.75 mL (instead of 1 mL) of acetonitrile were applied. Drying of the [^18^F]fluoride was performed with a stream of nitrogen under reduced pressure. A solution of the nitro-precursor in dimethylsulfoxide (0.5 mg/0.3 mL) was added to the dried [^18^F]F^−^/K_2_CO_3_/Kryptofix complex and heating was performed at 140 °C for 10 min to afford the nucleophilic substitution reaction and to remove the Boc-protecting group. The crude radiolabeling mixture was diluted with 3 mL of a 1 to 2 dilution of preparative mobile phase in water for injection (WFI) and purified using reverse-phase HPLC (RP-HPLC) on an XBridge C_18_ column (5 μm, 4.6 × 150 mm; Waters, Milford, MA, USA), eluted with a mixture of sodium phosphate buffer 0.01 M pH 7.4 and acetonitrile (75/25 v/v) at a flow rate of 1 mL/min and with UV detection at 263 nm. [^18^F]AV1451 eluted around 21 min, was collected in a vial filled with 15 mL WFI and passed over C_18_ Sep-Pak cartridge (Waters; activated with 5 mL ethanol (EtOH) and 10 mL WFI). After a rinse with NaCl 0.9%, [^18^F]AV1451 was eluted from the Sep-Pak with 1.6 mL EtOH, followed by a rinse with 17.4 mL NaCl 0.9% and sterile filtered (Millex-GV filter 0.22 μm ø 13 mm) into the final batch vial. Assessment of the radiochemical and chemical purity and identity confirmation were performed using RP-HPLC on an XBridge column (C_18_, 3.5 μm, 3.0 × 100 mm; Waters) eluted with a mixture of sodium phosphate buffer 0.01 M pH 10.5 and acetonitrile (75/25 v/v) at a flow rate of 0.8 mL/min. UV detection was performed at 263 nm. [^18^F]AV1451 and was synthesized with a radiochemical purity of > 98% and an average molar activity of 959 GBq/μmol (*n* = 13) at the end of synthesis.

In general, [^18^F]THK5351 was synthesized according to previously reported procedures [40] in remote controlled home-made R&D synthesis modules by a nucleophilic fluorination of the tosyl-protected THK5352 precursor, followed by acid hydrolysis and neutralization. [^18^F]fluoride was produced, separated from the ^18^O-water, eluted from the anion exchange cartridge and azeotropically dried with acetonitrile as described by Declercq et al. [39]. A solution of the precursor in dimethylsulfoxide (0.5 mg/0.25 mL) was added to the dried [^18^F]F^−^/K_2_CO_3_/Kryptofix complex and heating was performed at 110 °C for 10 min to afford the nucleophilic substitution reaction. Removal of the tetrahydropyranyl protecting group was accomplished with 2 N HCl (0.3 mL) at 90 °C for 3 min followed by neutralization with 4 N NaOAc (0.36 mL). The crude radiolabeling mixture was purified using RP-HPLC on an XBridge C_18_ column (5 μm, 4.6 × 150 mm; Waters) eluted with mixtures of sodium phosphate buffer 0.01 M pH 7.4 and EtOH (72/28 v/v) at a flow rate of 0.8 mL/min and with UV detection at 226 nm. [^18^F]THK5351 was eluted around 24 min. The purified radiotracer solution was diluted with saline to obtain an EtOH concentration < 10% suitable for intravenous injection. Quality control was performed on an XBridge column (C_18_, 3.5 μm, 3.0 × 100 mm; Waters) eluted with a mixture of sodium phosphate buffer 0.01 M pH 6 and acetonitrile (78/22 v/v) at a flow rate of 0.8 mL/min. UV detection was performed at 226 nm. [^18^F]THK5351 was synthesized with a radiochemical purity of > 98% and an average molar activity of 161 GBq/μmol (*n* = 10) at the end of synthesis.

### Semi-quantitative phosphor screen autoradiography

Brain tissue was sectioned at the time of brain removal, following standard anatomic landmarks. Areas of interest were identified and frozen at − 80 °C. For each patient, a piece of the anterior part of the inferior temporal/occipitotemporal gyrus was removed from the frozen tissue slab available from the right hemisphere (Supplementary Fig. 1a). This coordinate is based on the in vivo signal on tau-PET studies in SV PPA [13] (Supplementary Fig. 1b). Consecutive 20 μm-sections were cut in a cryostat (− 20 °C), mounted on glass slides, and stored at − 20 °C until use. Adjacent cryosections were used for both autoradiographical and immunohistochemical studies for optimal anatomical matching.

Cases were divided into three sets so that each of the three storage phosphor screens contained one SV PPA case, as well as a positive (AD) and a negative control (HC). The in vitro [^18^F]AV1451 binding study was performed according to the methods described by Xia et al. [41] and Declercq et al. [39] with some minor modifications. The air-dried 20 μm cryosections of the right anterior part of the inferior temporal gyrus were incubated with 15 kBq [^18^F]AV1451 (640 GBq/μmol; 200 μL per section; 0.1 pM) at room temperature for 60 min and subsequently washed at room temperature with mixtures of phosphate-buffered saline (PBS) and EtOH (1 min in 0.01 M PBS pH 7.4-2 min in 0.01 M PBS pH 7.4/EtOH 30/70 v/v-1 min in 0.01 M PBS pH 7.4/EtOH 70/30 v/v-1 min in 0.01 M PBS pH 7.4) [17, 41]. To assess specificity of binding, sections were incubated with 200 μL of radiotracer in the presence of 10 μM of authentic (“cold”) AV1451 compound (self-block). Coincubation with 10 μM of deprenyl was performed to assess off-target binding to MAO-B enzyme [27]. For all cases, one section was included for each test condition (tracer *n* = 1/tracer + cold *n* = 1/tracer + deprenyl *n* = 1) except for the SV PPA cases for which two sections were incubated with tracer solution (tracer *n* = 2/tracer + cold *n* = 1/tracer + deprenyl *n* = 1). All sections were incubated with tracer prepared from a single production, excluding effects of differences in molar activity. After drying of the slices, autoradiograms were obtained by overnight exposure to a phosphor storage screen (super-resolution screen; Perkin Elmer, Waltham, USA), read using a Cyclone Plus system (Perkin Elmer) and semi-quantitatively analyzed using Optiquant software (Perkin Elmer). Results were obtained as digital light units per square mm (DLU/mm^2^). Semi-quantitative analysis of binding was performed by calculating the ratio of bound [^18^F]AV1451 over bound [^18^F]AV1451 in presence of 10 μM AV1451 and the ratio of bound [^18^F]AV1451 over bound [^18^F]AV1451 in presence of 10 μM deprenyl. As a preliminary, exploratory analysis, an identical procedure was applied in a subset of cases (case 4, 6, 8, and 11) (Table 1) for [^18^F]THK5351 to assess translatability of findings to another first-generation tau-PET tracer (molar activity [^18^F]THK5351 199 GBq/μmol; 0.4 pM). Adjacent cryosections were stained with phospho-tau (AT8), and pTDP-43 (phospho-Ser409/410 TDP-43 as described in the previous section. The degree of astrogliosis was analyzed using Glial fibrillary acidic protein (GFAP) immunohistochemistry (polyclonal rabbit, 1:500, DAKO Z033401–2, Agilent Technologies Heverlee, Belgium) and the degree of activated microglia (microgliosis) was assessed using CD68 (monoclonal mouse, 1:50, DAKO M081401–2, Agilent Technologies Heverlee, Belgium). A DAKO Autostainer (Universal Staining System Carpinteria, California) was used to perform immunohistochemical stainings. After this reaction, the tissue was counter-stained with hematoxylin, and posterior incubation with DAB. Non-specific signals such as endogenous peroxidase were blocked to prevent false positive results. Sections were also stained with Perl’s staining to visualize iron content as potential off-target binding [20] and with the Gallyas silver impregnation technique to identify NFTs.

For each case, semi-quantitative burden was assessed by a single neuropathologist (DRT) for AD-tau, FTLD-tau, and FTLD-TDP (Table 1).

## Results

**Fig. 1:**
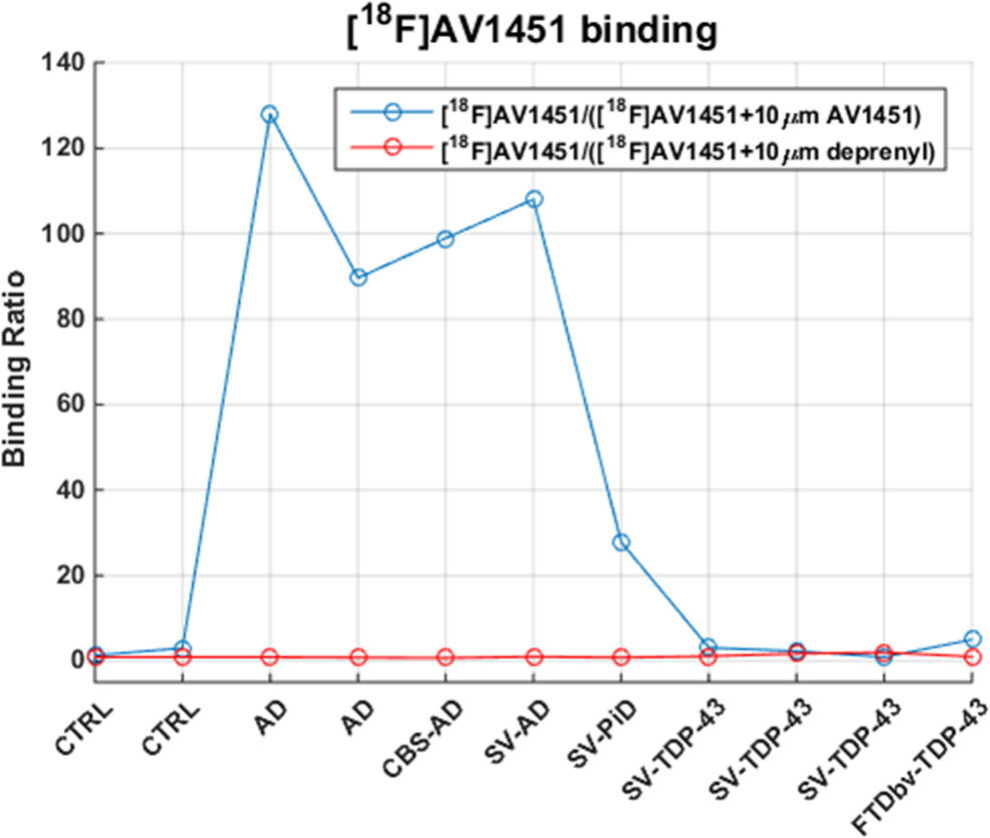
Semi-quantitative analysis of the in vitro binding of [^18^F]AV1451 on brain cryosections of different subjects. Ratio of total [^18^F]AV1451 binding (DLU/mm^2^) over [^18^F]AV1451 binding in the presence of 10 μM cold AV1451 (DLU/mm^2^) (blue) and ratio of total [^18^F]AV1451 binding (DLU/mm^2^) over [^18^F]AV1451 binding in the presence of 10 μM deprenyl (DLU/mm^2^) (red). The sequence of values follows the order of subjects in Table 1

A summary of the demographic characteristics and pathologic classification of the different cases is shown in Table 1. Results of the in vitro [^18^F]AV1451 autoradiography binding study are presented in Fig. 1.

### In vitro [^18^F]AV1451 binding in neuropathological defined cases

The two negative control subjects were free of AD tau pathology in the inferior temporal cortex (both Braak stage I; Table 1, cases 1 and 2) and served as internal negative controls. These controls did not show any specific cortical [^18^F]AV1451 binding (Fig. 1).

All four cases with underlying AD pathology had a Braak stage VI, reflecting severe and widespread AD tau pathology. Considering [^18^F]AV1451 binding, the two typical AD cases (cases 3 and 4) showed the expected pattern, with strong cortical [^18^F]AV1451 binding, which was almost completely blocked in the presence of the authentic reference material (“cold” compound) and this result served as an internal positive control. The specific binding was 51 times stronger than the signal measured in controls.

Also for the SV PPA-AD case (case 6) and the CBS-AD case (case 5), a strong cortical [^18^F]AV1451 binding, which was blocked in presence of cold AV1451, was observed and was situated in the range of the typical AD cases.

The SV PPA due to PiD (case 7) had no AD tau pathology (Braak NFT stage 0) but showed severe FTLD-tau pathology. The degree of astrogliosis and microgliosis was moderate, which was a similar finding as in the cases with underlying AD. This PiD case showed also substantial cortical [^18^F]AV1451 binding, which could be blocked by coincubation with the cold compound. The specific signal was 13 times higher than the average signal in controls, but 4 times lower than the specific signal in typical AD.

The three cases with SV PPA due to FTLD-TDP (cases 8–10) showed milder AD tau pathology compared with the cases with underlying AD pathology (Table 1: Braak NFT stage I-II vs. VI). Consequently, no NFTs were observed in the inferior temporal/occipitotemporal gyrus of these cases.

None of the SV PPA due to FTLD-TDP cases showed cortical [^18^F]AV1451 binding. Even the most severely affected case (case 8), with amyloid phase 2, Braak NFT stage II (transentorhinal cortex AD tau), and severe TDP-43 pathology as well as severe astrogliosis and microgliosis, did not show specific cortical [^18^F]AV1451 binding. The observed binding was in the same range of the non-AD/non-FTLD controls, and cortical [^18^F]AV1451 binding in SV-FTLD-TDP cases was on average 52 times lower compared with binding in the typical AD cases.

The FTD-bv case (case 11) had NFT pathology restricted to the medial temporal lobe (Braak NFT stage III). This case did not show clear cortical [^18^F]AV1451 binding, a finding which was similar to the SV PPA FTLD-TDP cases. For cases with specific binding (AD and PiD), no deprenyl effect was seen. Furthermore, as none of the FTLD-TDP cases showed cortical [^18^F]AV1451 binding, deprenyl did not affect the signal.

None of the studied cases showed iron deposition on Perl’s blue stainings that could potentially explain off-target binding (Table 1).

To evaluate whether the observed in vitro binding properties of [^18^F]AV1451 are shared with other first-generation tau-PET tracers also showing consistently elevated binding in SV PPA cases in vivo [13], we performed a similar autoradiography binding experiment with [^18^F]THK5351 in a subset of cases (case 4, 6, 8, and 11) (Table 1). We determined the ratio of bound [^18^F]THK5351 over bound [^18^F]THK5351 in presence of 10 μM cold ligand to assess specificity of binding. A similar binding pattern was observed with [^18^F]THK5351 as for [^18^F]AV1451: specific binding in cases with underlying AD (SV PPA (case 6); ratio 6.9 and typical AD (case 4); ratio 3.7), with absence of binding in FTLD cases with underlying TDP-43 type C (SV PPA (case 8); ratio 1.3 and FTD-bv (case 11; ratio 2.2)). No negative control data were available for [^18^F]THK5351. Binding ratios were significantly lower for [^18^F]THK5351 (< 7) compared with [^18^F]AV1541 (range 90–130).

### Representative cases demonstrating [^18^F]AV1451 binding versus pathological substrates

**Fig. 2:**
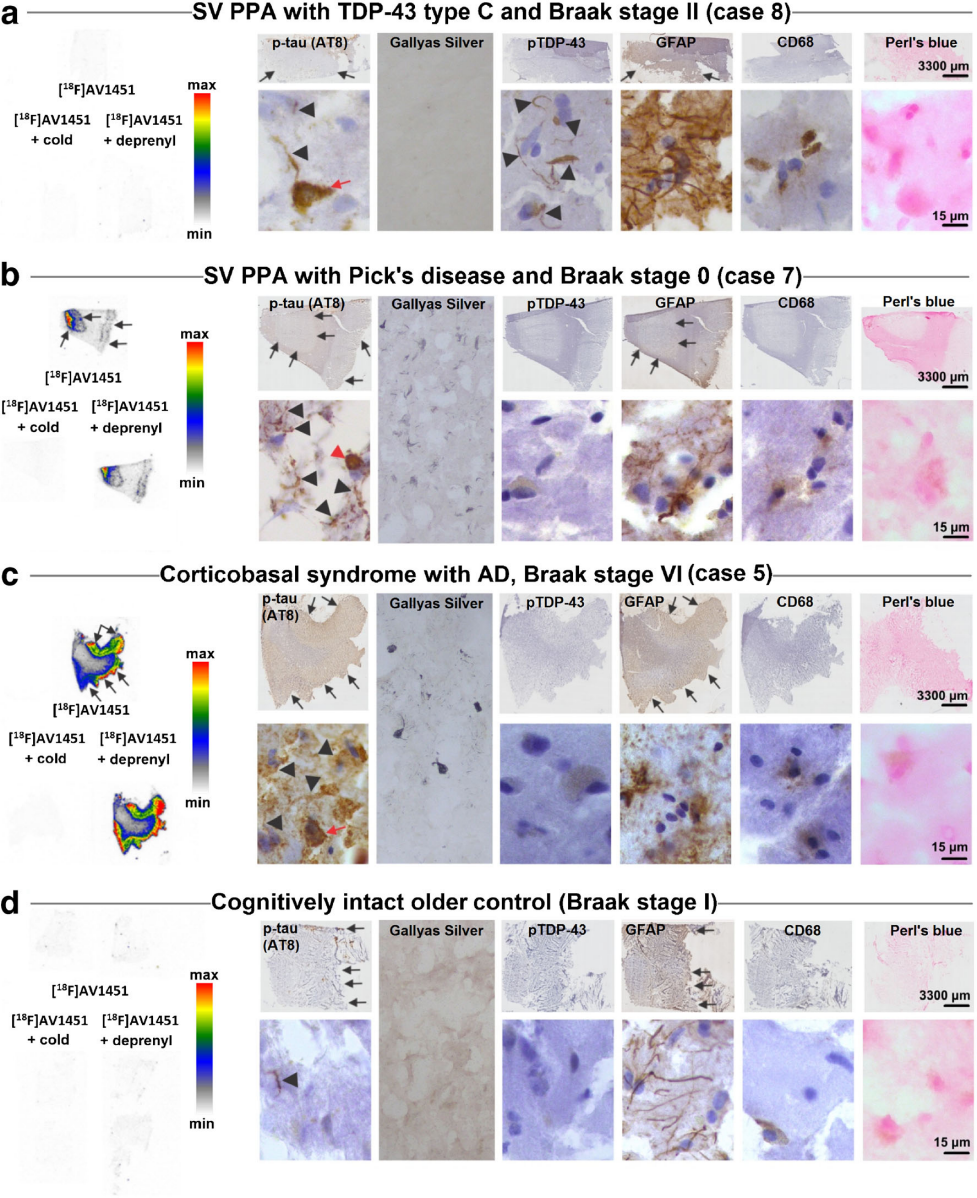
[^18^F]AV1451 in vitro binding versus pathological substrates on cryosections of the anterior part of the inferior temporal/occipitotemporal gyrus in representative cases. Tracer binding was studied, either in the presence of 10 μM AV1451 (cold; self-block) or in 10 μM deprenyl (MAO-B inhibitor). For each set of sections, tracer binding was normalized to the highest signal per phosphor storage screen (i.e., the most positive control on each of the three screens) and is visualized by a semi-quantitative color scale. **a** SV PPA with TDP-43 type C pathology (Case 8). Autoradiography shows no visible tracer binding. On immunohistochemistry, arrowheads point to p-tau positive neurites (AT8) or TDP-43 positive long dystrophic neurites (pTDP-43). The red arrow shows a single neurofibrillary tangle. GFAP shows abundant astrogliosis and CD68 indicates some microgliosis. Perl’s blue staining did not show abundant iron deposits. **b** SV PPA with Pick’s disease (Case 7). Autoradiography shows visible [^18^F]AV1451 binding, which is blocked by the cold compound but not affected by deprenyl. A p-tau positive Pick body is visible on AT8 staining (red arrowhead) and some neuropil threads (arrowheads). pTDP-43 was negative, GFAP showed some astrogliosis and CD68 indicated only a few microglia. Perl’s blue was negative. **c** CBS with AD pathology (Case 5). Autoradiography shows visible [^18^F]AV1451 binding, which is fully blocked by the cold compound. No visible displacement of tracer binding by deprenyl is observed. A p-tau neurofibrillary tangle is visible on AT8 staining (red arrow) and some neuropil threads (arrowheads). pTDP-43 was negative; GFAP showed some astrogliosis and CD68 indicated only a few microglia. Perl’s blue was negative. **d** Cognitively intact older control. Autoradiography shows no visible binding. Some neuropil threads are seen on AT8 and some mild astrogliosis on GFAP

Figure 2 shows a comparison between the autoradiographical binding patterns and adjacent immunohistochemically stained cryosections in representative cases.

The SV PPA case with TDP-43 type C pathology showed no visible [^18^F]AV1451 binding on the autoradiography image (Fig. 2a). Immunohistochemistry demonstrated severe TDP-43 type C pathology, characterized by pTDP-43 positive long dystrophic neurites. The AD tau pathology was limited to the transentorhinal cortex (Braak stage II) and thus did not overlap with the region used to assess [^18^F]AV1451 binding.

For the SV PPA with PiD (Fig. 2b), autoradiography showed visible [^18^F]AV1451 binding, which could be fully blocked by the cold compound but was not affected by deprenyl. P-tau positive Pick bodies were visible on the AT8 staining and neuropil threads were seen in regions with [^18^F]AV1451 binding. There was no AD tau pathology in this case (Braak stage 0) (Fig. 2b).

For the CBS case with underlying severe AD pathology (Fig. 2c), autoradiography showed strong [^18^F]AV1451 binding, which was completely blocked by the cold compound. No visible blocking effect of deprenyl was observed. P-tau NFTs and some neuropil threads colocalized with [^18^F]AV1451 binding.

The control case did not show any specific tracer binding and tau pathology was mild and restricted to the hippocampus (Braak stage I) (Fig. 2d).

## Discussion

We and others have previously demonstrated strong PET signal of the first-generation tau-PET tracers in the anterior temporal lobe that was consistently present in patients with SV PPA [9–16]. In an effort to explain this unexpected but highly consistent finding, we conducted an in vitro autoradiography binding study with [^18^F]AV1451 in SV PPA cases, constituting the range of underlying pathologies. Binding was compared with negative controls and to typical AD, corticobasal syndrome (CBS) due to neuropathological AD, and FTD behavioral variant (FTD-bv) due to TDP-43 type C. In all three cases with SV PPA due to FTLD-TDP, no specific [^18^F]AV1451 binding was observed. The absence of binding in controls as well as the successful blocking with authentic AV1451 in cases with tauopathy suggested the specificity of the [^18^F]AV1451 signal for tau. The specific [^18^F]AV1451 binding was highest in AD tau, followed by PiD tau. This binding colocalized with the respective tau lesions on immunohistochemistry. A subset of cases was assessed with [^18^F]THK5351, indicating similar results.

The absence of binding in the vitro autoradiography images of FTLD-TDP cases is in line with a recent study in which in vitro [^3^H]AV1451 autoradiography on frontal and temporal cortical cryosections of SV PPA cases as well as *C9orf72* TDP-43 type B cases also showed no binding [12]. Another independent study demonstrated the absence of in vivo [^18^F]AV1451 PET signal during life in a patient with the *C9orf72* mutation who was neuropathologically diagnosed as TDP-43 type B [21]. Incidental co-pathology of scattered NFTs in the middle frontal and inferior temporal gyrus showed corresponding mild [^18^F]AV1451 binding but without additional uptake matching the widespread TDP-43 type B pathology [21]. Apart from two earlier studies suggesting low binding to TDP-43 type A and C [20, 23], these findings, together with the current results, suggest that [^18^F]AV1451 does not bind to TDP-43 aggregates in FTLD-TDP. Although, as demonstrated in Tsai et al. 2018 [21], it cannot be excluded that some FTLD-TDP cases might have some tau pathology; the anterior temporal lobe localization of PET signal does not correspond to the expected distribution of tau in AD [31].

Moreover, in an independent study, strong anterior temporal lobe [^18^F]AV1451 binding was found in all seven cases with either SV PPA or “right” semantic dementia [11] despite four of these being AD-biomarker negative. In another study, all seven SV PPA cases had elevated anterior temporal lobe [^18^F]AV1451 binding but only one case appeared to be amyloid-positive on PET [10]. A recent in vivo PET study demonstrated that when SV PPA cases were stratified based on amyloid status, all 13 amyloid-negative SV PPA cases showed increased left anterior temporal cortex [^18^F]AV1451 binding, which was higher than the signal in typical AD [14]. If amyloid-positive, the SV PPA cases also showed, besides peak binding in left anterior temporal cortex, more widespread cortical binding than the amyloid-negative SV PPA cases [14]. In these studies, no post mortem information was available on these cases. Nevertheless, it seems highly unlikely that the amyloid-negative SV PPA cases are characterized by underlying AD based on the available amyloid-PET data.

Given that the degree of frontotemporal atrophy in FTD is related to the degree of astrocytic apoptosis [26], we expected that SV PPA with FTLD-TDP would be characterized by extensive astrocytic apoptosis and astrogliosis. The latter becomes the overwhelming pathological feature as the disease progresses [26]. More specifically, we expected that the strong [^18^F]AV1451 signal seen in vivo would relate to underlying astrogliosis, overexpressing MAO-B [42]. Semi-quantitative assessment of pathology demonstrated, however, that all patients included in this study (including all SV PPA) showed a similar degree of neuroinflammation (i.e., astrogliosis and microgliosis), regardless of the underlying neuropathological diagnosis. Moreover, no clear overlap was seen between neuroinflammatory markers and specific cortical [^18^F]AV1451 signal on the adjacent cryosection. This leaves us with an open question regarding the binding target that is present in vivo but possibly no longer active in postmortem sections, e.g., mediated by reactive astrocytes or microglia. We speculate that target binding may require living astrocytes or microglia, e.g.,if transmembrane transporters or enzymatic modifications are involved. However, knowledge on the specific nature of these other binding targets of AV1451 is currently limited. Alternatively, the target may be present in the inflammatory reaction during an early disease stage but not in the end stage of the disease. For instance, in the AD dementia stage, neocortical in vivo binding of [^11^C]deuterium-L-deprenyl is not increased compared with controls, while in the MCI stage [^11^C]deuterium-L-deprenyl binding is significantly increased [27]. We used patient tissue of cases that are end-stage disease. Therefore, the absence of MAO-B binding might not only depend on the spatial pattern of normal MAO expression [43] but also on the temporal dynamics of disease-related processes such as gliosis. In a post mortem study using AD brain tissue, significantly higher binding was observed in the temporal lobes and in the white matter, which coincided with the presence of an increased number of activated astrocytes (on GFAP immunohistochemistry). Critically, the highest binding was observed in Braak NFT stage I-II, whereas it decreased with increasing Braak NFT stages [44]. A similar mechanism might occur in SV PPA in vivo, in which there could be an increase in MAO-B expression associated with astrogliosis, followed by a decrease in MAO-B expression when end stage disease has been reached. Like [^18^F]AV1451, [^18^F]THK5351 PET also shows a very strong anterior temporal signal in SV PPA in vivo [13], which co-localizes with atrophy [10, 13], indicating it might be relevant to the pathophysiology associated with SV PPA.

Another source of potential off-target binding besides MAO, might relate to neuromelanin-containing cells, calcifications, or iron deposits as previously reported [20, 22, 24, 45, 46]. However, we could not demonstrate an overlap between autoradiographic binding and iron deposits in the current study.

Furthermore, we should be aware that in vivo settings might differ from in vitro settings. For example, in Parkinson’s disease, use of MAO-B inhibitors at pharmaceutical levels did not significantly affect [^18^F]AV1451 binding in vivo [47]. This made the authors conclude that MAO-B does not appear to be a significant binding target of [^18^F]AV1451, despite tau levels being low in this patient group in the first place. Similarly, we also could not reveal any binding in vitro while in vivo, a strong signal has been consistently shown [9–12, 14]. On the other hand, for [^18^F]THK5351, in vivo reduction of tracer uptake has been demonstrated in AD and PSP using an oral dose of selegiline [25]. In in vitro autoradiography studies, deprenyl at concentrations of 150 nM and 500 nM, was able to reduce [^18^F]THK5351 binding on AD sections of the striatum, but also on sections of the prefrontal cortex and hippocampus [25]. We did not observe a significant effect of coincubation with deprenyl on [^18^F]AV1451 binding in the AD cases. The anterior temporal lobe has generally low MAO-B expression levels in healthy conditions [43]. Therefore, it cannot be excluded that other regions apart from the anterior temporal lobe would have shown an effect of deprenyl in AD. As discussed earlier, another caveat is that in our study, MAO-B levels might be generally low [44] since the selected AD and PiD cases were end-stage disease.

Differences in results can also relate to the use of brain sections as used here versus brain homogenates as used in some of the other studies. When brain sections of cases that demonstrated specific binding (i.e., AD and PiD cases) were incubated with a mixture of high molar activity [^18^F]AV1451 and a high concentration of the MAO-B inhibitor deprenyl, no reduction in tracer binding was observed in the current study. This indicates that MAO-B binding cannot explain the source of off-target binding in these cases. In contrast, a previous in vitro study of Lemoine et al. demonstrated a reduction in [^3^H]deprenyl binding in homogenates of AD patients when a competition assay was performed with unlabeled AV1451 [48]. In contrast to the current in vitro autoradiography study, binding studies with [^3^H]AV1451 on AD brain homogenates showed 10% displacement by the MAO-B inhibitor deprenyl and 80% (*K*_i_ = 0.70 nM) by the MAO-A inhibitor clorgyline [24]. Also in non-AD brain frontal cortex homogenates, [^3^H]AV1451 binding was completely inhibited with nanomolar affinity (*K*_i_ = 0.43 nM) by the MAO-A inhibitor clorgyline [49]. The [^3^H]AV1451 binding affinity for MAO was comparable to its affinities for aggregated tau [24] and as such could lead to a confounding signal in PET studies. Of note is that the latter study showed that there might be regional differences in these off-target proteins. In the temporal cortex, the [^3^H]AV1451 binding is essentially sensitive to clorgyline but not to deprenyl, indicating a majority of binding to MAO-A in the temporal cortex. Given that the temporal cortex is high in MAO-A levels [43], [^18^F]AV1451 may also bind to MAO-A in vivo [24, 50]. However, since no binding was observed in the SV PPA cases of our autoradiography experiment, the MAO-A binding hypothesis as explanation for the observed signal is less credible.

The absence of in vitro binding in FTLD-TDP might indeed be due to discrepancies in methodology as previously suggested [22]. Firstly, numerical differences in the binding levels measured in AD compared with non-AD brains as detected by [^3^H]AV1451 in vitro binding on brain homogenates seem relatively narrow [22] when compared with the autoradiographic techniques that make use of brain sections that show a much higher signal-to-noise ratio. This may be due to the high ethanol concentrations (70-30%) used in the autoradiographic studies [17, 41], being much more effective at washing out nonspecific binding than the PBS washes used in the homogenate binding studies [19, 22, 24]. Moreover, in vitro autoradiography with [^18^F]THK5351 (an arylquinoline) equally showed absence of binding in FTLD-TDP cases. The absence of binding therefore does not seem to be attributable to an idiosyncratic interaction between ethanol washing and [^18^F]AV1451. Processing of the tissue might result in complex physicochemical interactions that may alter the secondary structure of tau fibrils and possibly, also the structure of TDP-43 aggregates. First, the use of brain sections versus brain homogenates might affect the accessibility of the tracer to the binding sites on the tau fibrils. Secondly, the use of ethanol may also affect the accessibility of the binding target due to its potential to denaturate proteins and given that ethanol is not naturally present in the brain [51–53]. Therefore, we cannot exclude that [^18^F]AV1451 and [^18^F]THK5351 are binding to TDP-43 aggregates in vivo despite the clear absence of binding in post mortem studies.

In the SV PPA case with underlying PiD, we observed in vitro [^18^F]AV1451 binding which colocalized with tau pathology and this tracer binding could be blocked by the cold compound, suggesting specific binding to FTLD tau. The binding signal was, however, four times lower than the average signal in AD. This is interesting given that in PiD, tau fibrils exist as straight filaments composed out of 3 repeat (3R) tau while [^18^F]AV1451 is designed to have high affinity for the paired helical fragment structure of AD which is constituted out of 3R/4R tau [7]. In the autoradiography study of Lowe et al. on paraffin-embedded formalin fixed tissue [20], binding to PiD was suggested but not semi-quantified and was also lower in PiD than in AD cases. Sander et al. showed moderate [^18^F]AV1451 binding on cryosections of a PiD case and displaceable binding in the 4R tauopathies CBD and PSP [23]. These findings are in contrast with the study of Marquie et al. [19], in which no detectable [^18^F]AV1451 binding was demonstrated in cryosections containing FTLD-tau lesions from PiD (and other tauopathies: PSP and CBD) when the signal was contrasted to that of control brains. In the study of Tsai et al. [21], a patient with sporadic bvFTD demonstrated punctate inferior temporal and hippocampus tracer retention, corresponding to the area of severe AGD pathology, a 4R tauopathy. This case also had Braak stage III [21]. Overall, these findings, together with our current results, indicate low affinity of [^18^F]AV1451 for FTLD tau.

## Limitations

In the current study, a semi-quantitative approach was applied to analyze the in vitro binding results. However, no affinity constants could be derived, which would have been more accurate. Also, a direct comparison between in vivo and in vitro binding was not possible as none of the cases received tau-PET during life. Nevertheless, in vivo binding in the left anterior temporal cortex in amyloid-negative SV PPA is relatively consistent between subjects both for [^18^F]AV1451 and for [^18^F]THK5351 [9–16] and therefore it seems likely that the SV PPA cases used in the current study would have shown a similar in vivo PET pattern.

Overall, the sample size is low, which is partly due to the low prevalence of PPA in general [2]. Nonetheless, our results demonstrate that [^18^F]AV1451 also binds to some extent to FTLD tau and not only to AD tau. Although we and others demonstrated that [^18^F]AV1451 does not bind to TDP-43 pathology in vitro, this tracer can still be of diagnostic value. Compared with currently available diagnostic imaging tools, the discriminative ability of [^18^F]AV1451 in PPA is as high as that of [^18^F]FDG PET [9] and higher than that of MRI [54]. However, since the in vivo binding target of the first generation tau-PET tracers in non-AD dementias remains currently unknown, these tracers cannot be used as a reliable biomarker.

The current study findings illustrate the lack of understanding of in vivo [^18^F]AV1451 and [^18^F]THK5351 binding to pathological proteins in SV PPA and possibly also in other diseases, but could not resolve the discrepancy between in vivo and in vitro findings.

## Conclusion

In vitro autoradiography on frozen brain sections revealed the absence of [^18^F]AV1451 and [^18^F]THK5351 binding in SV PPA due to FTLD-TDP, while clear binding was present in SV PPA due to underlying tauopathies (AD and PiD). We speculate that the discrepancy between the in vitro autoradiography findings and the high in vivo PET signal in SV PPA relates to a target that is present in vivo but no longer active in postmortem sections. Familiarity with distinct types of “off-target” binding is required before clinical use of [^18^F]AV1451 can take place [53].

## Electronic supplementary material

Supplementary Fig 1 (DOCX 651 kb)
