# Supplementary material for: Binding of [18F]AV1451 in post mortem brain slices of semantic variant primary progressive aphasia patients
Source: Eur J Nucl Med Mol Imaging. 2019 Dec 18;47(8):1949–60. doi: 10.1007/s00259-019-04631-x (PMC7300115; doi:10.1007/s00259-019-04631-x)
Supplement: Supplementary file 1 — (DOCX 651 kb) [file 259_2019_4631_MOESM1_ESM.docx]

**
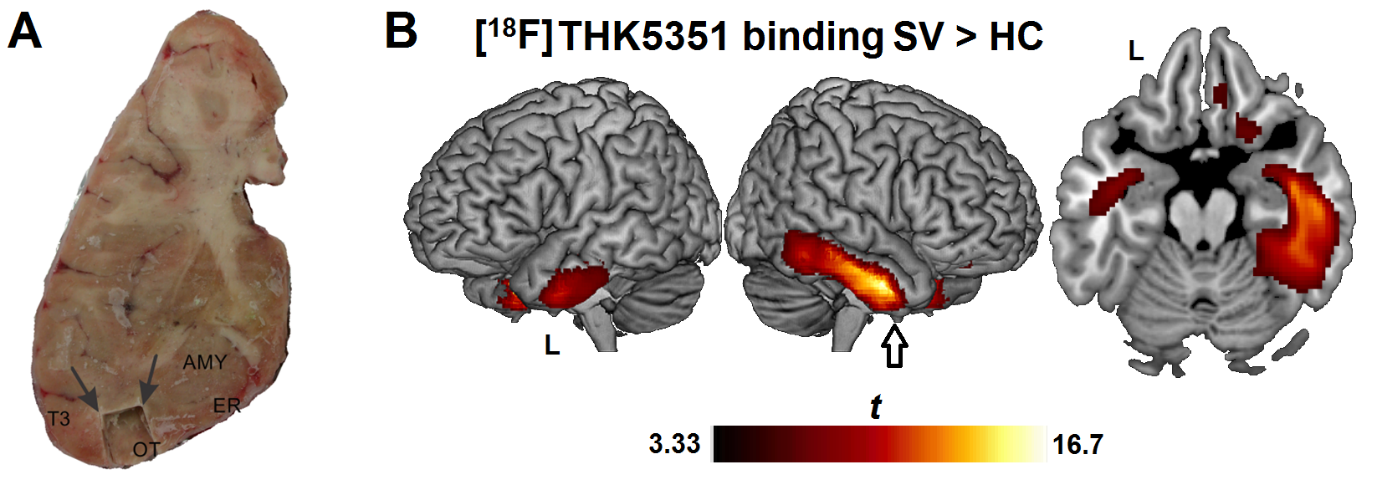
**

**Supplementary Fig 1.** (**a**) Sampling position of the anterior part of the inferior temporal/occipitotemporal gyrus. (**b**) This coordinate is based on the in vivo signal seen in tau-PET studies in SV PPA contrasted to healthy controls (HC). Figure adapted from [13] with permission of reprint. AMY = amygdala, ER = entorhinal cortex, OT = occipitotemporal gyrus, T3 = third temporal sulcus.
